# Text mining in a literature review of urothelial cancer using topic model

**DOI:** 10.1186/s12885-020-06931-0

**Published:** 2020-05-24

**Authors:** Hsuan-Jen Lin, Phillip C.-Y. Sheu, Jeffrey J. P. Tsai, Charles C. N. Wang, Che-Yi Chou

**Affiliations:** 1 Department of Biomedical Informatics, Asia University, 500, Lioufeng Rd., Wufeng, Taichung, Taiwan; 2 Division of Nephrology, Asia University Hospital, Taichung, Taiwan; 3 Kidney Institute and Division of Nephrology, China Medical University Hospital, Taichung, Taiwan; 4 Department of Electrical Engineering and Computer Science, University of California, Irvine, 5200 Engineering Hall, Irvine, CA 92697 USA; 5 Department of Post-baccalaureate Veterinary Medicine, Asia University, Taichung, Taiwan; 6 Department of internal medicine, Asia University Hospital, Taichung, 413 Taiwan

**Keywords:** Urothelial carcinoma, Text mining, Topic modeling, LDA2vec, Research trends

## Abstract

**Background:**

Urothelial cancer (UC) includes carcinomas of the bladder, ureters, and renal pelvis. New treatments and biomarkers of UC emerged in this decade. To identify the key information in a vast amount of literature can be challenging. In this study, we use text mining to explore UC publications to identify important information that may lead to new research directions.

**Method:**

We used topic modeling to analyze the titles and abstracts of 29,883 articles of UC from Pubmed, Web of Science, and Embase in Mar 2020. We applied latent Dirichlet allocation modeling to extract 15 topics and conducted trend analysis. Gene ontology term enrichment analysis and Kyoto encyclopedia of genes and genomes pathway analysis were performed to identify UC related pathways.

**Results:**

There was a growing trend regarding UC treatment especially immune checkpoint therapy but not the staging of UC. The risk factors of UC carried in different countries such as cigarette smoking in the United State and aristolochic acid in Taiwan and China. GMCSF, IL-5, Syndecan-1, ErbB receptor, integrin, c-Met, and TRAIL signaling pathways are the most relevant biological pathway associated with UC.

**Conclusions:**

The risk factors of UC may be dependent on the countries and GMCSF, IL-5, Syndecan-1, ErbB receptor, integrin, c-Met, and TRAIL signaling pathways are the most relevant biological pathway associated with UC. These findings may provide further UC research directions.

## Background

Urothelial carcinoma (UC) also known as transitional cell carcinoma includes carcinomas of the bladder, ureters, renal pelvis. UC is the fourth common cancer in men [1]. Risk factors of UC include cigarette smoking [2], chronic urinary tract inflammation, analgesics abuse, exposure to arylamines in the organic chemical, rubber, and paint and dye industries [3], Balkan nephropathy [4], chlorinated drinking water [5], arsenic-contaminated drink water [6], radiotherapy [7], and cyclophosphamide [8]. Non-muscle invasive bladder UC can be treated using transurethral bladder tumor resection and intravesical therapy [9]. Muscle-invasive bladder cancer is associated with a poor prognosis and is treated with neoadjuvant chemotherapy followed by cystectomy [10]. New treatment for UC such as immune checkpoint inhibitors is used for advanced and metastatic UC [11].

There is a large volume of publications on UC. Traditional ways of literature review tend to be time-consuming and labor-intensive. Machine-learning-based literature mining may analyze large collections of documents, identifies patterns in a dataset using statistical and computational methods, make predictions based on the discovered patterns, and minimizes human interventions. Machine learning has been used in biomedical informatics research and early prediction of treatment outcomes. Literature mining using machine learning is useful in summarizing key research themes and trends [12]. A topic model is a probability-based text mining approach to identify the topics and has been applied to literary analysis in many research fields [13]. In this study, we extract a set of topics from the abstract of UC using a topic model, analyze the dynamics of topics, and explore the biological pathways associated with UC.

## Methods

### Data set

We used the keyword “urothelial cancer” to search abstract from PubMed, Web of science, and Embase in Mar 2020. Fourteen thousand four hundred forty-three abstracts were obtained from Pubmed, 14,390 from Web of Science, and 24,110 from Embase. A total of 29,883 abstracts were analyzed after the removal of the duplicated ones. The title and abstract of each article were extracted and then combined into a single string. The keywords assigned by authors were not included [14]. The general words (such as background, aim, objective, purpose, method, result, conclusion), stop words, numerical digits, punctuation, and symbols were removed.

### Topic modeling

Latent Dirichlet Allocation (LDA) is a type of topic modeling. Lda2vec is an extension of word2vec and learns word, document, and topic vectors. LDA learns the powerful word representations in word2vec and constructs a human-interpretable LDA document. The LDA document is obtained by modifying the skip-gram variant. In the original skip-gram method, the model is trained to predict context words based on a pivot word. Lda2vec goes one step beyond the paragraph approach by working with document-sized text fragments and decomposing the document into two different components - a document weight vector and a topic matrix. The document weight vector represents the percentage of the different topics and the topic matrix consists of different topic vectors. A context vector is constructed by combining the different topic vectors in a document [15].

Lda2vec is an unsupervised text mining method and to determine the optimal number of topics is critical. There is no best way of choosing the optimal number of topics [16]. The perplexity measure may estimate the optimal number of topics, its result is difficult to interpret. The optimal number of topics is usually decided by researchers. We tested Lda2vec with 10, 15, and 20 topics, and compared the similarity and difference of content of topics obtained using the different models to determine the optimal number of topics.

### Visualization of topics

For visualization of the content of topics, the most probable words to convey a topic meaning were listed with the RGB color model, an additive color model in which red (R), green (G), and blue (B) light are added together in various parameters to reproduce a broad spectrum of colors. The parameters of R, G, and B are all inversely proportional to the normalized probability of words, and the color is shaded in greyscale from black to white. The higher color depth indicates a higher probability. The RGB color model was plotted with python (wordcloud package version 1.6.0). The word clouds were also plotted to demonstrate the distribution of vocabularies over each topic. To make the visualization clear, we combined the singular and the plural forms of a word as one word if both forms were listed in the top 20 probable words for a given topic. The topics were individually presented as an unstructured set of word clouds, and the word size is proportional to the probability of the word within a topic, *P (word|topic).*

### Gene ontology and pathway enrichment analysis

To investigate a comprehensive set of functional annotations of the hub gene. Gene Ontology (GO) term enrichment analysis and Kyoto Encyclopedia of Genes and Genomes (KEGG) pathway analysis were performed by using the “FunRich” [17]. FunRich is a functional enrichment and interaction network analysis tool, which allowed the updating database for performing functional enrichment analysis. GO enrichment analysis and KEGG pathway analysis were performed with the FunRich functional enrichment analysis tool (version 3.1.3). A *p*-value of < 0.05 was considered significant [18].

## Results

**Fig. 1:**
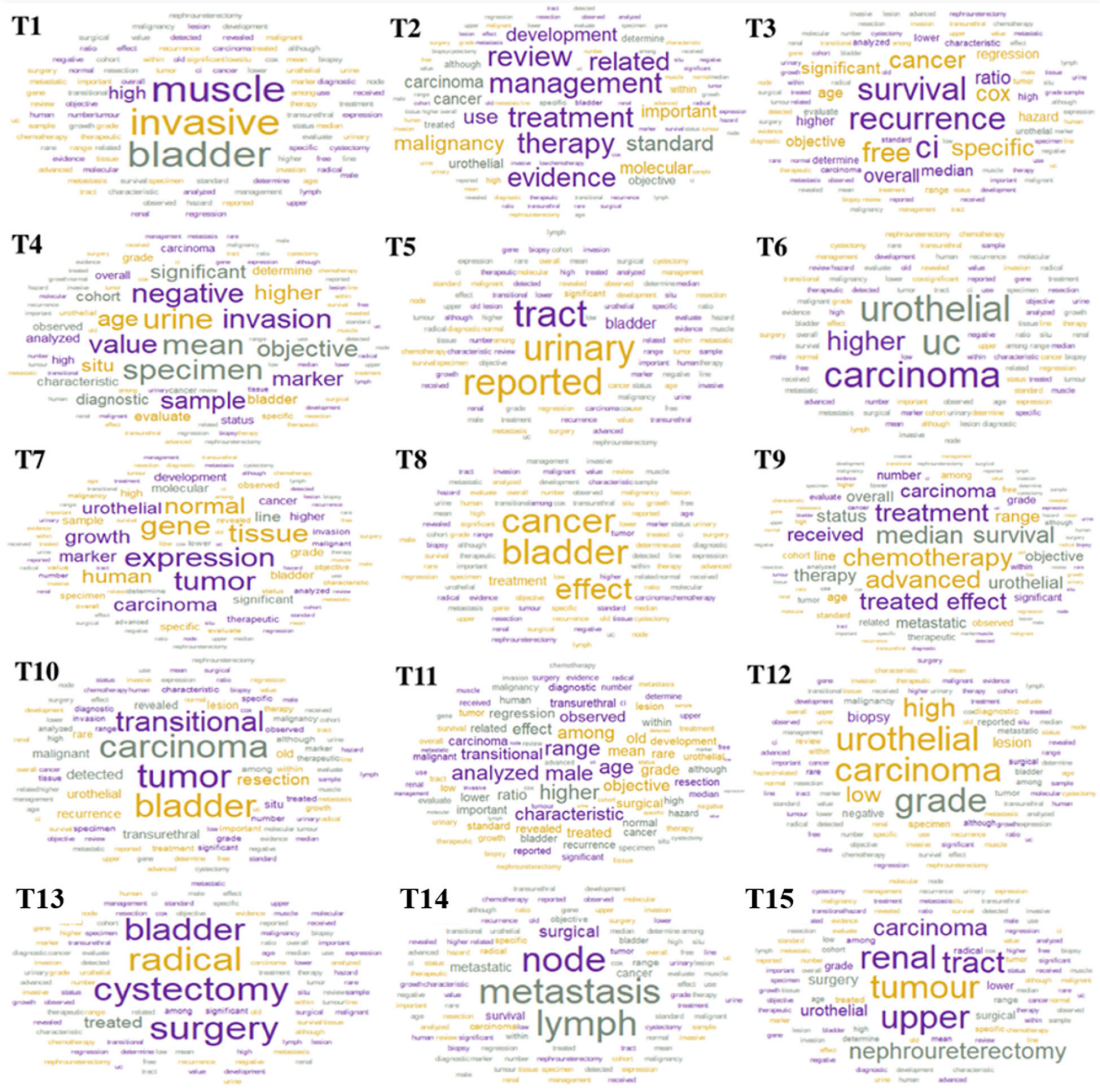
Word frequency clouds of 15 topics

We explored the top 10, 15, 30 keys words and selected the top 15 keywords. LDA discovered separate and relative definite issues, the location of UC (T5, T6, T8, T15), gene (T7), treatment (T2, T9, T10, T13, T15), and severity (T1, T4, T12, T14). Some of the topics are related. For example, the gene expression (T7) and tumor grade (T12) are associated with the decision of chemotherapy (T9), surgery (T10, T15), and survival (T3). The keywords in each topic are shown in Table 1. The word clouds of 15 topics (Fig. 1) provide better visualization of the topics. The larger font size depth indicates a higher probability of the word. Muscle, invasive, and bladder were the most frequent words in T1 because T1 was about the severity of UC. Muscle invasion of the urinary bladder was a key characteristic of advanced UC. Higher, urothelial, and carcinoma were the most frequent words that appeared in T6 because T6 is about upper urinary tract UC. As T14 is about metastatic UC, the most frequent words were metastasis, lymph, and node.

**Table 1:** The most probable keywords in 15 topics of LDA2vec

T1	Severity	invasive, muscle, bladder, high, cancer, tumor, significant, CI, overall, lower
T2	Treatment	treatment, therapy, management, review, evidence, related, standard, malignancy, use, development
T3	Survival	recurrence, survival, ci, free, cancer, specific, cox, overall, ratio, significant
T4	Urine	mean, urine, specimen, negative, invasion, value, sample, objective, age, higher
T5	Bladder	urinary, tract, reported, bladder, significant, urothelial, review, lower, among, revealed
T6	Upper urinary tract	UC, urothelial, carcinoma, higher, negative, upper, within, tract, tumor, characteristic
T7	Gene	expression, gene, tumor, tissue, normal, carcinoma, human, growth, urothelial, marker
T8	Lower urinary tract	bladder, cancer, effect, treatment, tumor, transurethral, among, detected, lower, number
T9	Chemotherapy	chemotherapy, median, advanced, treatment, treated, survival, effect, carcinoma, received, therapy
T10	Surgery	tumor, carcinoma, bladder, transitional, resection, detected, transurethral, urothelial, recurrence, malignant
T11	Patients’ characteristics	male, higher, range, analyzed, age, among, characteristic, transitional, objective, effect
T12	Grade	carcinoma, urothelial, grade, high, low, lesion, biopsy, negative, reported, specimen
T13	Radical cystectomy	cystectomy, radical, surgery, bladder, treated, among, significant, treatment, carcinoma, age
T14	Lymph Node metastasis	metastasis, node, lymph, surgical, metastatic, cancer, survival, range, carcinoma, radical
T15	Nephroureterectomy	tumour, renal, upper, tract, carcinoma, nephroureterectomy, urothelial, surgery, lower, grade

*UC* urothelial cancer

**Fig. 2:**
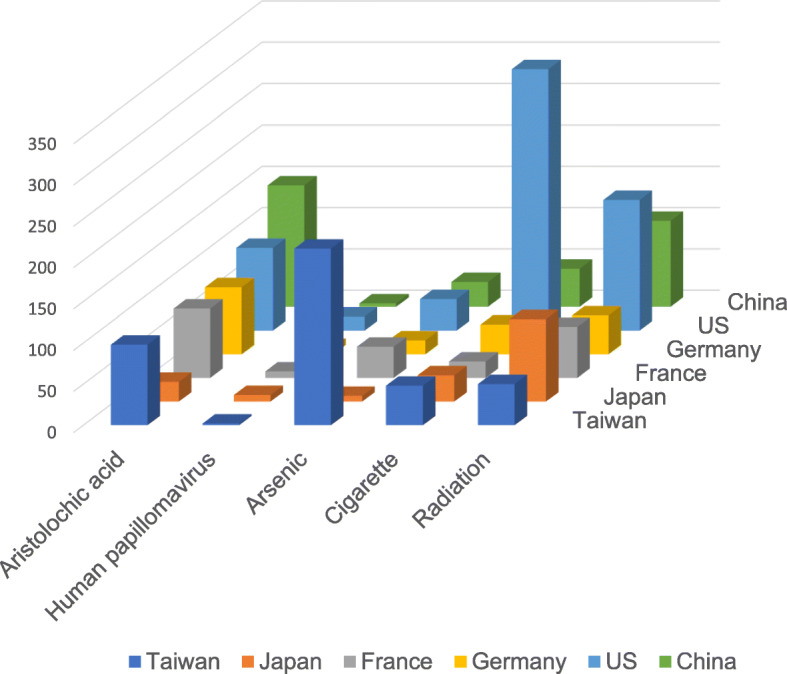
The number of publications according to risk factors and countries

There was an association between risk factors of UC and countries in the analysis of 13,725 abstracts (Fig. 2). The top 10 publications were from the United States, Taiwan, China, Germany, Japan, France, India, Italy, Span, and Iran. The top 10 risk factors of UC were cigarette, radiation, arsenic, aristolochic acid, human papillomavirus, chronic cystitis, cyclophosphamide, aromatic amines, coffee, and tea. Most of the studies reported the association between UC and aristolochic acid were from the United States, Taiwan, and China. Arsenic associated publications were mainly from Taiwan. Most publications focusing on risk factors such as cigarettes, human papillomavirus, and radiation are from the United States.

### Gene ontology and pathway enrichment analysis

**Fig. 3:**
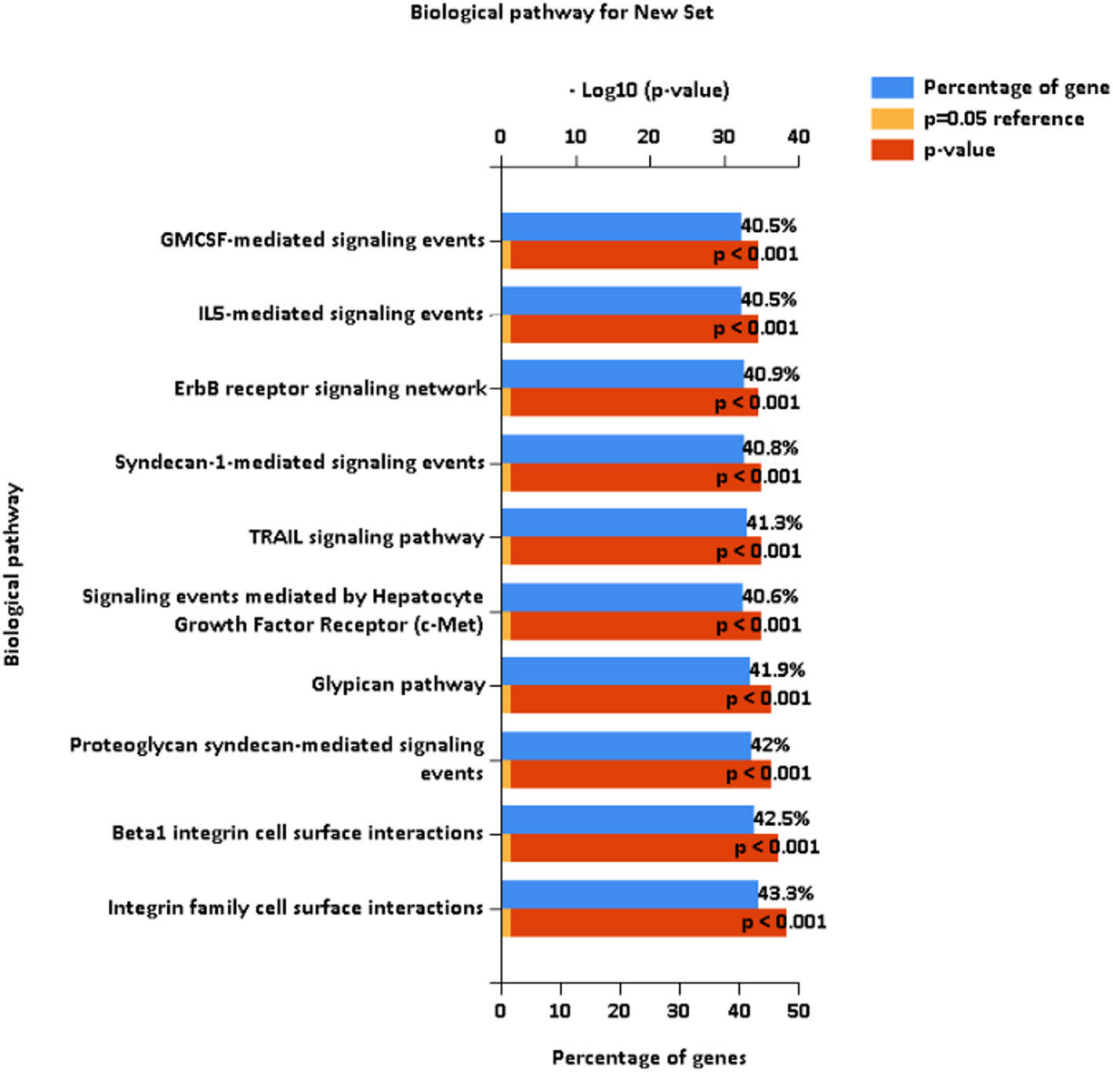
The top ten pathways in which urothelial cancer was significantly involved (ranked by *p*-value using the FunRich 3.0 software). A p-value < 0.05 was regarded as significant

A total of 15,491 abstracts were associated with genes related to UC and we identified the pathway according to the identified gene. The top ten pathways associated with UC were granulocyte-macrophage colony-stimulating factor (GMCSF)-medicated signal events, interleukin (IL) 5-mediated signaling events, ErbB receptor signaling network, Syndecan-1-mediated signaling events, TNF-related apoptosis-inducing ligand (TRAIL) signaling pathway, Signaling events mediated by Hepatocyte Growth Factor Receptor (c-Met), Glypican pathway, Proteoglycan syndecan-mediated signaling events, Beta1 integrin cell-surface interactions, and Integrin family cell surface interactions (Fig. 3). The percentage of the gene in the publications ranged from 40.5 to 43.3%. The pathways from top to bottom are listed according to the *P*-values of the hypergeometric test.

## Discussions

In this text mining assisted literature review of UC, we found an increasing trend of publications regarding treatment, survival, and gene. A decreasing trend of publications regarding upper urinary tract UC, radical cystectomy, and lymph node metastasis was also observed. Immune checkpoint therapy is the hottest topic in the UC treatment. The majority of the publications are from the United States, China, Japan, Taiwan, Germany, Italy, and France. Cigarette smoking and aromatic amines are commonly reported risk factors [19, 20], followed by radiation, arsenic, aristolochic acid, and human papillomavirus. Tea and coffee [21–23] have been also extensively studied in their association with UC and they have a neutral or beneficial effect on UC. Aristolochic acid is commonly used for urinary tract and respiratory tract infection in traditional Chinese medicine can be associated with renal failure and UC [24–26]. Most of the publications about aristolochic acid are from Taiwan and China. But many reports were from the United States, Germany, and France. This may suggest that exposure to aristolochic acid is common in Taiwan and China but is not limited to these countries. The difference in risk factors among different countries may suggest racial differences in cancer susceptibility and the importance of the environmental factor in the pathogenesis of UC.

The top ten pathways identified may help to explore new treatment for UC. One of the examples is Mycobacterium bovis bacillus Calmette-Guérin. Mycobacterium bovis bacillus Calmette-Guérin has been used as an effective treatment for UC because it activates the TRAIL signaling pathway that leads to tumor necrosis through the immune response [27]. GMCSF is associated with aggressive tumor cell growth [28]. IL5-mediated signaling and Syndecan-1-mediated signaling [29] enhances cancer cell migration and invasion [30]. ErbB receptor signaling [30] and cell-surface integrin [31] increases cancer cell resistance to chemotherapy. Hepatocyte Growth Factor Receptor (c-Met) [32] and glypican [33] are linked to the clinical outcomes. Medications that target these pathways may be used to treat UC.

There are some limitations to this study. First, only results from Pubmed were analyzed and the language is limited to English. This may lead to selection bias. Second, the analysis was conducted based on the extracted abstracts but not the full texts. More information may be obtained if we apply analysis on full texts. Third, we used LDA to extract articles. LDA was the concept of “bag of words” rather than the order of words. When a sentence was divided into separate words, it became meaningless or lost the original meaning. Forth, the frequency of words was presented but the frequency of the words may not necessarily stand for their significance.

## Conclusion

In this paper, we have presented an empirical study by utilizing LDA modeling to discover major research topics of UC. We analyzed the dynamics and intellectual structure of topics. We found growing researches on the treatment but not cancer staging. Cigarette smoking and arsenic are the most commonly reported risk factors worldwide and there is an association between UC risk factors and countries. GMCSF, IL-5, Syndecan-1, ErbB receptor, integrin, c-Met, and TRAIL signaling pathways are the top biological pathways associated with UC. The study provides a better understanding of the trends of UC research and potential future research directions.

## Data Availability

The datasets used and/or analysed during the current study are available from the corresponding author on reasonable request.

## Abbreviations

UC: Urothelial cancer
GMCSF: Granulocyte-macrophage colony-stimulating factor
IL-5: Interleukin-5
ErbB: Epidermal growth factor receptor family
c-Met: Tyrosine-protein kinase Met
TRAIL: TNF-related apoptosis-inducing ligand
LDA: Latent dirichlet allocation
